# Immediate effect of local vibration on motor unit firing behavior and muscle strength in healthy young adult males

**DOI:** 10.1007/s00421-024-05553-9

**Published:** 2024-08-07

**Authors:** Yuichi Nishikawa, Aleš Holobar, Kohei Watanabe, Toshihiko Komatsuzaki, Takanori Chihara, Jiro Sakamoto, Takashi Kawagoe, Hidetaka Nagayasu, Kyoko Mori, Kenji Kawano, Noriaki Maeda, Shinobu Tanaka, Allison S. Hyngstrom

**Affiliations:** 1 Faculty of Frontier Engineering, Institute of Science and Engineering, Kanazawa University, Kakuma-Machi, Kanazawa, 920-1192 Japan; 2 Faculty of Electrical Engineering and Computer Science, University of Maribor, Maribor, Slovenia; 3 Laboratory of Neuromuscular Biomechanics, School of Health and Sport Sciences, Chukyo University, Nagoya, Japan; 4 Faculty of Advanced Manufacturing Technology Institute, Kanazawa University, Kanazawa, Japan; 5 Department of Health and Psychosocial Medicine, Aichi Medical University School of Medicine, Nagakute, Japan; 6 Division of Interior Planning and Development, Toyota Boshoku Corporation, Toyota, Japan; 7 Division of Seat Evaluation and Engineering, Toyota Boshoku, Toyota, Japan; 8 Division of Sports Rehabilitation, Graduate School of Biomedical and Health Sciences, Hiroshima University, Hiroshima, Japan; 9 Department of Physical Therapy, Marquette University, Milwaukee, WI USA

**Keywords:** Vibration, Antagonist muscle, Motor unit, Electromyography, Vastus lateralis

## Abstract

**Purpose:**

The aim of this study was to examine the effect of vibration on motor unit (MU) firing behavior and physical performance of antagonist muscles in healthy young adult males.

**Methods:**

Fourteen males (age = 24.3 ± 3.6 years) were included in this study. There were two conditions, one in which participants received 80 Hz vibration in the distal tendon of the hamstring for 30 s and the control condition (no vibration). High-density surface electromyography (HD-SEMG) signals and maximal voluntary contraction (MVC) of knee extensor muscles were evaluated before and after the respective conditions and recorded from the vastus lateralis muscle during submaximal ramp-up and sustained contractions at 30% MVC. Convolution blind source separation was used to decompose the HD-SEMG signals into individual MU firing behaviors.

**Results:**

In total, 739 MUs were detected (control; 360 MUs and vibration; 379 MUs), and a total of 312 matched MUs were identified across both submaximal contraction conditions (control: 150 MUs; vibration: 162 MUs). Vibration significantly increased the discharge rate (*p* = 0.047) and decreased the recruitment threshold before and after intervention (*p* = 0.001) but not in the control condition. Furthermore, the recruitment threshold is a factor that influences discharge rate. Significant correlations were observed between the recruitment threshold and both the ∆ discharge rate and the ∆ recruitment threshold under the vibration condition (*p* < 0.001).

**Conclusion:**

Vibration increased in the discharge rate and decreased the recruitment threshold of the antagonist muscle. These findings suggested that vibration contributes to immediate changes in the neural control of antagonist muscles.

## Introduction

Vibration has emerged as a potential adjunct to strength training. An increase in muscle strength may be optimized after the application of local vibration (Alghadir et al. 2018). Vibration is an attractive intervention because local vibration is well tolerated, effective and easy to use; in addition, local vibration is known to be a beneficial intervention technique not only for healthy individuals but also for those with neurodegenerative disorders (Murillo et al. 2014). However, studies thus far have focused on the vibration of the agonist muscle and the influence of localized vibration on muscle strength and neural activity based on the excitatory, monosynaptic Ia afferent pathway (Romaiguère et al. 1993). When the vibrated muscle contracts, the classic motor response is the tonic vibration reflex (TVR) (Goodwin et al. 1972). The TVR activates motoneurons through a polysynaptic projection of the primary afferents of the spindle (Romaiguère et al. 1991). However, the effects of vibration of the antagonist muscle on the agonist muscle have still not been explored.

In healthy adults, an accompanying excitatory tonic response (antagonistic vibration response, AVR) is observed in the antagonist muscle of the muscle tendon receiving vibration intervention (Roll et al. 1980). Vibration is thought to inhibit the contraction of antagonist muscles via Ia inhibitory interneurons. However, previous studies have reported that tendon vibration results in increased muscle activity in the antagonist muscle (Feldman and Latash 1982; Rothmuller and Cafarelli 1995). This phenomenon may have been caused by increased excitation of Renshaw cells due to vibration exciting alpha motoneurons, thus inhibiting Ia inhibitory interneurons and increasing their coactivation (Rothmuller and Cafarelli 1995). Furthermore, several previous studies have reported that vibration of the agonist muscle increases corticospinal excitability in the antagonist muscle, as measured by motor-evoked potential amplitudes using transcranial magnetic stimulation in young adults (Forner-Cordero et al. 2008; Talis et al. 2010). Although there is evidence of changes in spinal, cortical and corticospinal pathway excitability, it is not clear how vibration affects motor unit (MU) firing behavior and muscle strength in the antagonist muscle.

The purpose of this study was to determine whether vibration of the biceps femoris tendon (antagonist) has an excitatory effect on the activation of MU firing during submaximal ramp-up and sustained contractions and on the muscle strength of the quadriceps muscle (agonist). A previous study reported that stimulation of the patellar tendon resulted in increased hamstring activation (Rothmuller and Cafarelli 1995); however, a different stimulation site was chosen for the present study. Based on these findings, we hypothesized that, in terms of reciprocal innervation, inhibition of the vibrated muscle (biceps femoris muscle) would result in activation of the antagonist muscle (vastus lateralis (VL) muscle), which would lead to increased muscle strength and an increased MU discharge rate. To help us interpret our results, we assessed MU firing behavior and knee extensor strength using high-density surface electromyography (HD-SEMG) and dynamometer data.

## Methods and materials

### Participants

Fourteen healthy young males (age = 24.3 ± 3.6 years, height = 172.2 ± 4.8 cm, weight = 60.9 ± 6.6 kg) were included in this study after their written informed consent was obtained (Table 1). The inclusion criteria were independence in daily life, no history of orthopedic diseases, and no need for assistive devices when walking. The exclusion criteria were a diagnosis of neuromuscular diseases, cardiovascular diseases, or diabetes mellitus. This study was approved by the Ethics Committee of the Institute of Science and Technology, Kanazawa University, in accordance with the Declaration of Helsinki (approval no. 2021–5).

**Table 1:** Characteristics of participants

	Healthy young males (*n* = 14)
Age, year	24.3 ± 3.6
Height, cm	172.2 ± 4.8
Weight, kg	60.9 ± 6.6
Thickness of subcutaneous tissue, mm	4.8 ± 1.4
Knee extension torque, Nm	181.8 ± 44.8

Data are shown as the mean ± standard deviation

### Experimental procedures

**Fig. 1:**
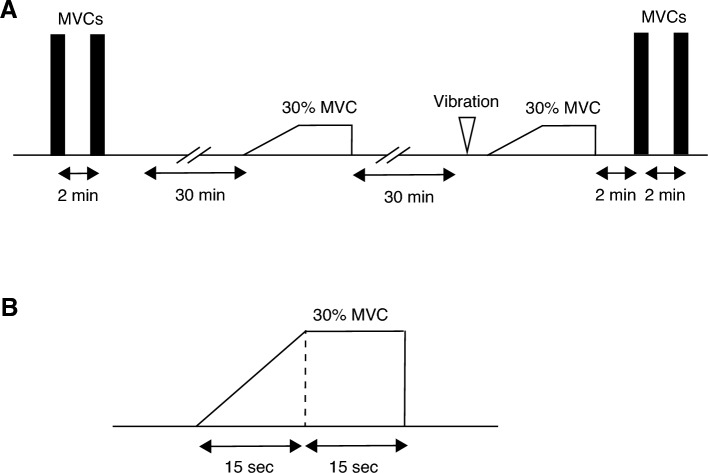
Summary of the study protocol. **A** Participants performed maximum voluntary contraction (MVC) of the knee extensor muscles, followed by a submaximal contraction task of up to 30% MVC. After the submaximal contraction task was completed, the participants were moved to a custom-made seat where the vibration intervention was performed. After the intervention, the participants performed the submaximal contraction task, and MVC measurements were taken again. **B** Participants were asked to perform ramp-up and hold contraction tasks (ramp up to 30% MVC in 15 s and sustained contraction for 15 s). The RMS was calculated from 1 s of the midpoint of the sustained contraction

All participants were asked to perform isometric maximum voluntary contractions (MVCs) of the test limb of the knee extensor muscles (on the kicking side). A custom-made dynamometer (Takei Scientific Instruments Co., Ltd., Niigata, Japan) was used to acquire MVC data. During contraction, the angles of the hip and knee joints were positioned at 90°. All participants completed two MVC trials, and there was a pre evaluation warm-up period of ten minutes that included indoor walking and lower-limb stretching (Fig. 1). The target torque for the submaximal ramp-up contractions was calculated from the peak MVC torque generated at baseline. After recording the MVC measurements, all participants performed a submaximal isometric ramp-up contraction from 0 to 30% MVC (Watanabe et al. 2016) (participants were to ramp up to 30% MVC in 15 s and sustain the contraction for 15 s; Fig. 1). The target torque and the torque generated by a participant were displayed on a monitor. Electromyography (EMG) data coinciding with MVC tasks and submaximal ramp-up contraction tasks were evaluated. To verify the immediate effect of vibration stimulation on muscle strength, the MVC was also measured immediately after the vibration intervention. At least 30 min were allowed between the MVC task and the submaximal ramp-up task to minimize the potential effects on the decline in contractile function (Fig. 1A). The EMG signals remained affixed throughout the experiment. When participants were moving to the seat for the vibration experiments, movement of the knee joint was avoided as much as possible. Measurements in the vibration and control conditions were acquired at least 1 day apart to exclude crossover effects.

### Local vibration stimulation and control conditions

**Fig. 2:**
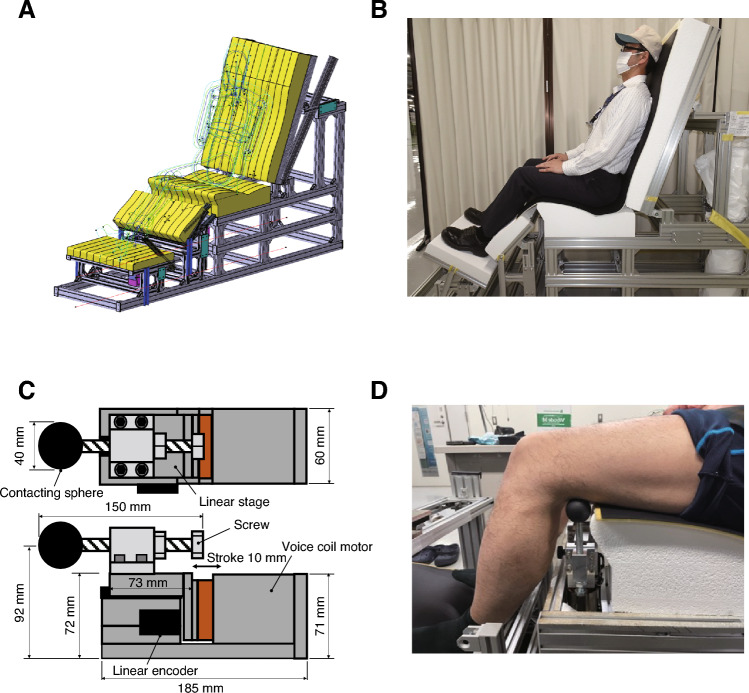
Custom-made seat and vibration device. The seat was made of rigid styrene foam (**A**). Participants were instructed to sit on the seat while in a resting posture (**B**). A voice coil motor was used for the custom-made vibration device (**C**). Vibration was applied directly to the distal biceps femoris muscle tendon (**D**)

The subjects sat on a custom-made seat made of rigid Styrofoam and maintained a resting position (Fig. 2A and B). Vibration was applied to the test limb (kicking side) of the biceps femoris tendon with a custom-made vibrator (Voice Coil Motor, AVM60-HF-10, Akribis Systems Japan, Tokyo, Japan) (Fig. 2C and D). The vibration conditions were as follows: frequency, 80 Hz; duration, 30 s; and amplitude, 0.1 mm. In the control condition, participants were placed in the same position sat for the same amount of time. Participants were instructed to keep their muscles relaxed throughout the vibration and control conditions. The tester monitored muscle activity by visual observation.

### EMG recording

HD-SEMG signals were recorded from the VL muscle using a 64-channel electrode array (GR08MM1305, OT Bioelettronica), consisting of 13 rows and 5 columns (electrodes were 1 mm, diameter; there was a distance of 8 mm between electrodes in each direction, and one electrode was missing at the upper left corner) according to the same procedure used in previous studies (Watanabe et al. 2012; Nishikawa et al. 2017a, b). According to previous studies, the electrode grid was placed on the VL muscle, and the center of the grid was located between the lateral edge of the patella and the head of the greater trochanter (Nishikawa et al. 2017b, 2018, 2021). After the skin was cleaned (80% alcohol), a biadhesive sheet (KIT08MM1305, OT Bioelettronica) with conductive paste (Elefix ZV-181E, NIHON KOHDEN, Tokyo, Japan) was used to attach the electrode grid to the muscle surface (Nishikawa et al. 2017b). A ground electrode was placed on the patella, whereas a reference electrode for VL grids was placed on the fibular head. A 16-bit analog-to-digital converter (Quattrocento, OT Bioelettronica, sampling frequency of 2048 Hz) was used to record the monopolar HD-SEMG signals. The signals were amplified with a gain of 150 and off-line bandpass-filtered at 10–500 Hz. MATLAB software (MATLAB 2021b, MathWorks GK, MA, USA) was used to analyze the force exerted and EMG signals.

### Data processing

Based on adjacent electrodes, 59 bipolar EMG signals were calculated (12 bipolar recordings in each column except for the far right column, which had 11 electrode pairs). The root mean square (RMS) was calculated for each bipolar signal, and the mean values were calculated for all the bipolar signals. Furthermore, the RMS of the MVC was calculated from 1 s of data centered on the maximum torque data during the MVC. The RMS of the 30% MVC condition was calculated as the 1-s rolling RMS average from the sustained contraction and normalized to the RMS of the MVC (Fig. 1B). HD-SEMG recordings were separated into individual MU discharge timings using a validated convolutive blind source separation technique (Holobar and Zazula 2004; Merletti et al. 2008; Holobar et al. 2009). Individual MUs were identified using DEMUSE software (v. 6.0; The University of Maribor, Slovenia). Interspike intervals below 4 Hz or above 30 Hz (Holobar et al. 2009; Watanabe et al. 2016), as well as low-quality signals (pulse-to-noise ratio < 30 dB (MU firing identification accuracy < 90%) (Holobar et al. 2014), were excluded. From the time interval between spikes, we calculated the instantaneous discharge rates of identified MUs (pulses per second, pps). The coefficient of variation (CV) for the interspike interval was defined as the ratio between the standard deviation and the mean value of the interspike interval. In each identified MU, the mean discharge rate was calculated during sustained contractions (15 s, Fig. 1B). Further analysis did not include MUs with CVs greater than 30% during the submaximal ramp-up task or during sustained contractions (Fuglevand et al. 1993).

Previous reports have acknowledged that the recruitment of additional MUs with untracked activity may have confounded comparisons of MU firing behavior between time points (Bigland-Ritchie et al. 1992; Christova et al. 1998). Therefore, in the present study, MU filters were estimated from the decomposition of signals acquired during one contraction (i.e., preintervention) and applied to signals acquired during the contraction postvibration, as described in a previous study (Frančič and Holobar 2021). The filter was also applied to MU tracking, ensuring an accuracy of > 90% for MU firing identification during submaximal ramp-up contraction to 30% MVC based on a pulse-to-noise ratio > 30. The knee extension torque, RMS of MVC, MU discharge rate, and recruitment threshold were calculated from pre- and postintervention values, and the ∆ knee extension torque, ∆ RMS of MVC, ∆ discharge rate, and ∆ recruitment threshold were calculated.

### Statistical analysis

The data are presented as the mean ± standard deviation or median (min–max). The Shapiro–Wilk test was performed to confirm the normality of the data. The significant differences in knee extension torque and normalized RMS were analyzed using repeated-measures analysis of variance (ANOVA) for the two groups (condition [vibration and control] $\times$ period [pre- and postintervention]). Differences between each group and period were analyzed using Bonferroni post hoc correction.

An analysis of the mean discharge rate and recruitment threshold (including all identified MUs and only the matched MUs) was conducted using a mixed-effects model (containing a random effect for subject and fixed effect for condition (vibration and control) and period (preintervention and postintervention). In the analysis of the mean discharge rate, the recruitment threshold was included as a covariate. Means and 95% confidence intervals (CIs) for the discharge rate and recruitment threshold in each condition were estimated.

Correlations of the recruitment threshold with the ∆ discharge rate and ∆ recruitment threshold of only matched MUs were analyzed using repeated-measures correlation coefficients (rmcorr package of R).

The Shapiro‒Wilk test, ANOVA and mixed-effects model analysis were performed with Stata version 17 (Stata Corp LLC, Texas, USA). Repeated-measures correlation coefficients were calculated with R, version 4.1.0 (R Foundation for Statistical Computing, Vienna, Austria), and graphs were generated with GraphPad Prism version 8 (GraphPad Software Inc., California, USA). Statistical significance was defined as *p* < 0.05.

## Results

### Knee extensor torque

**Fig. 3:**
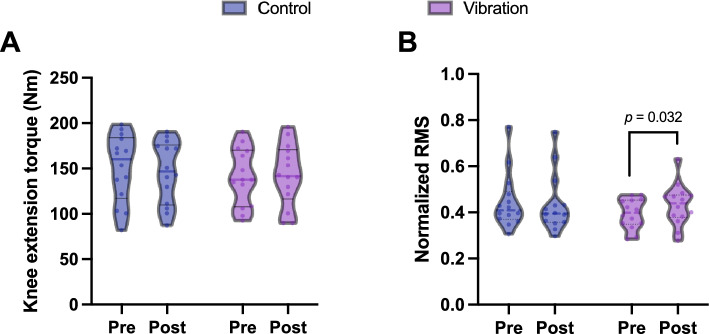
Comparison of the MVC and normalized RMS data. **A** There was no significant difference in the MVC between the control and vibration conditions. **B** RMS was calculated from the 30% MVC task and normalized to the RMS of the MVC. A significantly greater normalized RMS after the intervention than before the intervention was observed in the vibration condition but not in the control condition

The knee extension torque was calculated from the MVC values measured in the before and after conditions. Knee extension torque did not show a significant condition $\times$ period interaction (*F* = 2.488, *p* = 0.1268; *η*^2^ = 0.09) and did not show a significant difference between period (*F* = 0.4570, *p* = 0.5050, *η*^2^ = 0.02) and group (*F* = 0.1147, *p* = 0.7376, *η*^2^ = 0.004) (Fig. 3A).

### Normalized RMS

The normalized RMS was calculated by normalizing the RMS value from the 30% MVC task to the RMS value from the MVC task.

The normalized RMS showed a significant interaction of condition $\times$ period (*F* = 8.16, *p* = 0.0083, *η*^2^ = 0.238). The normalized RMS was significantly greater after intervention than before intervention in the vibration condition (*p* = 0.032, average differential value = 0.037, 95% CI = 0.008 to 0.067) but not in the control condition (*p* = 1.000, average differential value = –0.012, 95% CI = −0.041 to 0.017) (Fig. 3B).

### Detected *MUs*

MU firing behavior was calculated from muscle activity recorded while participants performed the 30% MVC task.

We detected a total of 739 MUs (control condition: 360 Mus and vibration condition: 379 MUs) and identified a total of 312 matched MUs across both submaximal contractions (control condition: 150 MUs and vibration condition: 162 MUs) that were considered for further analysis. The pulse-to-noise ratio of the obtained MUs before and after intervention was 33.41 ± 2.44 for both the control and vibration conditions.

### All detected *MUs*

The mean discharge rates and recruitment thresholds before and after intervention under control and vibration conditions are shown in Table 2.

**Table 2:** Mean and 95%CIs of discharge rate and recruitment threshold in all detected motor units

All detected MU	Adjusted mean discharge rate	Mean differences (vs. before)
Control		
Discharge rate		
Before	10.50 (9.85, 11.16)	
After	10.32 (9.66, 10.98)	−0.18 (−0.42, 0.05)
Recruitment threshold		
Before	18.08 (17.01, 19.16)	
After	16.95 (15.84, 18.07)	−1.13 (− 2.34, 0.08)
Vibration		
Discharge rate		
Before	10.02 (9.37, 10.67)	
After	10.42 (9.77, 11.08)	0.40 (0.18, 0.63)
Recruitment threshold		
Before	18.55 (17.52, 19.59)	
After	17.75 (16.64, 18.85)	−0.81 (−1.97, 0.36)

Data are presented as mean and 95% confidence interval

Adjusted for recruitment threshold

**Fig. 4:**
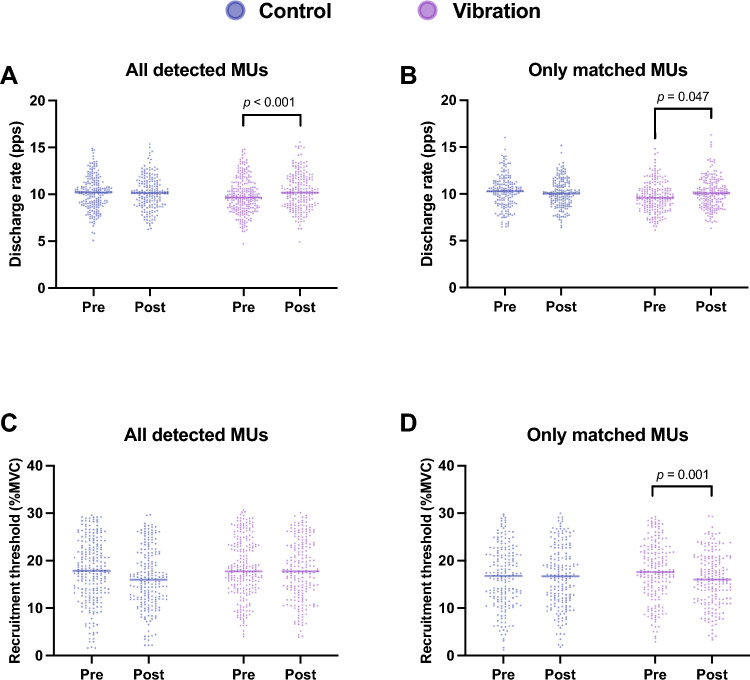
Comparisons of the discharge rates and recruitment thresholds of all detected motor units vs. only the matched motor units. A significantly greater discharge rate for all detected motor units (**A**) and only matched motor units (**B**) was observed in the vibration condition than in the control condition. The recruitment threshold of only matched motor units was significantly lower in the vibration condition than in the control condition (**D**). The results of two-way analysis of variance showed that the recruitment thresholds of all the detected conditions did not show a significant condition $\times$ period interaction (**C**)

A significantly greater mean discharge rate after intervention than before intervention was observed in the vibration condition (*p* < 0.001, average differential value = 0.40, 95% CI = 0.18 to 0.63 pps) but not in the control condition (*p* = 0.119, average differential value = –0.18, 95% CI = −0.42 to 0.05 pps) (Fig. 4A). Furthermore, the recruitment threshold included in the covariates was significant (*p* < 0.001).

Moreover, there was no significant difference in the recruitment threshold before and after the intervention in any condition (vibration, *p* = 0.175, average differential value = −0.81, 95% CI = −1.97 to 0.36% MVC; control, *p* = 0.068, average differential value = −1.13, 95% CI = −2.34 to 0.08% MVC).

### Matched *MUs* only

The mean discharge rates and recruitment thresholds before and after intervention under control and vibration conditions are shown in Table 3.

**Table 3:** Mean and 95%CIs of discharge rate and recruitment threshold in only matched motor units

All detected MU	Adjusted mean discharge rate	Mean differences (vs. before)
Control		
Discharge rate		
Before	10.53 (9.92, 11.13)	
After	10.33 (9.73, 10.94)	−0.19(−0.41, 0.02)
Recruitment threshold		
Before	16.78 (15.55, 18.00)	
After	17.01 (15.78, 18.24)	0.23 (−0.99, 1.45)
Vibration		
Discharge rate		
Before	9.94 (9.34, 10.54)	
After	10.15 (9.55, 10.76)	0.21 (0.00, 0.42)
Recruitment threshold		
Before	17.85 (16.63, 19.07)	
After	15.91 (14.69, 17.14)	−1.94 (−3.13, –0.75)

Data are presented as mean and 95% confidence interval

Adjusted for recruitment threshold

A significantly greater mean discharge rate after the intervention than before the intervention was observed in the vibration condition (*p* = 0.047, average differential value = 0.21, 95% CI = 0.00 to 0.42 pps) but not in the control condition (*p* = 0.077, average differential value = −0.19, 95% CI = −0.41 to 0.02 pps) (Fig. 4B). Furthermore, the recruitment threshold included in the covariates was significant (*p* < 0.001).

A significantly lower recruitment threshold after the intervention than before the intervention was noted in the vibration condition (*p* = 0.001, average differential value = −1.94, 95% CI = −3.13 to −0.75% MVC) but not in the control condition (*p* = 0.708, average differential value = 0.23, 95% CI = −0.99 to 1.45% MVC).

### Repeated-measures correlation coefficients

**Fig. 5:**
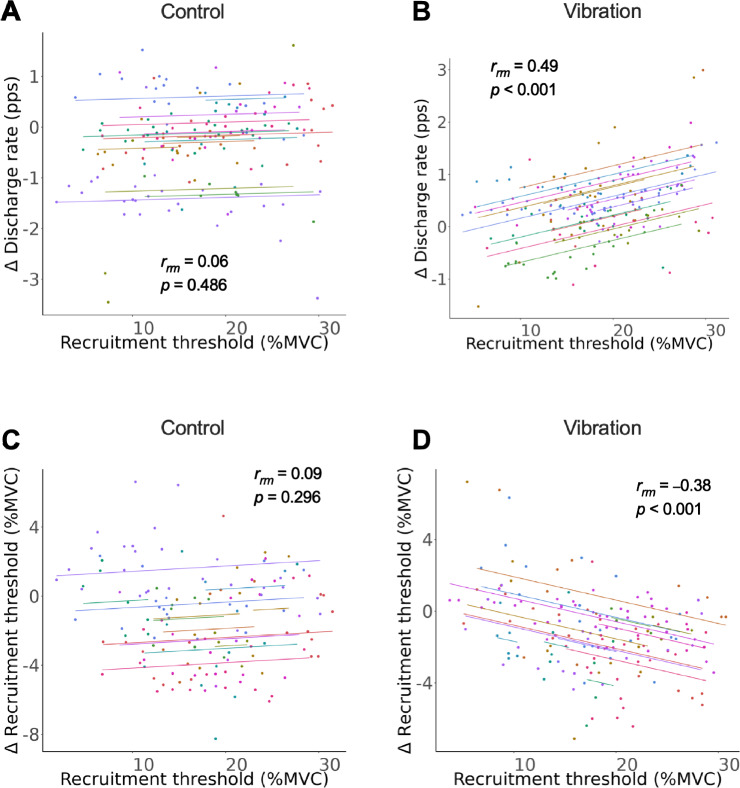
Repeated measures correlation coefficients of the recruitment threshold with the ∆ discharge rate and ∆ recruitment threshold in the control (**A**, **C**) and vibration conditions (**B**, **D**). Significant correlations were observed between the recruitment threshold and the ∆ discharge rate (**B**) and the recruitment threshold and the ∆ recruitment threshold (**D**) under the vibration condition. On the other hand, there was no significant correlation between the recruitment threshold and ∆ discharge rate (**A**) or between the recruitment threshold and the ∆ recruitment threshold (**C**) in the control condition

Significant correlations were detected between the recruitment threshold and the ∆ discharge rate (*r*_*rm*_ = 0.49, *p* < 0.001; Fig. 5B) and between the recruitment threshold and the ∆ recruitment threshold (*r*_*rm*_ =  − 0.38, *p* < 0.001; Fig. 5D) in the vibration condition but not in the control condition (*r*_*rm*_ = 0.06, *p* = 0.486; Fig. 5A and *r*_*rm*_ = 0.09, *p* = 0.296; Fig. 5C). These results showed that vibration had a greater impact on MUs recruited at higher thresholds than on those recruited at lower thresholds.

## Discussion

The purpose of this study was to compare the influence of vibration on the MU discharge rates of the antagonist muscle in young adult males during a 30% MVC task. Our novel results were as follows: (1) vibration at 80 Hz for 30 s contributed to activation of the MU of the antagonist muscle of the stimulated muscle, and (2) MUs recruited at higher thresholds were more affected by vibration than MUs recruited at lower thresholds during submaximal voluntary contraction (30% MVC). On the other hand, significant differences in the discharge rate and recruitment thresholds of MUs were not detected under control conditions. These findings partially supported our hypothesis. However, no changes in muscle strength due to vibration were observed.

Vibration has been shown to have both facilitative and suppressive effects on muscle spindle activity, both of which lead to altered motor output (Barrera-Curiel et al. 2019). The effect of vibration on motor output is believed to depend on the duration of the vibration. When the vibration at a frequency of 80 Hz was longer than 30 s, the discharge rate of the spindle of the stimulated muscle decreased in most of the Ia fibers derived from several leg muscles (Roll et al. 1989; Ribot-Ciscar et al. 1998). Recently, a study reported that 30 min of local vibration induced significantly decreased discharge rates of MUs and persistent inward current (PIC) magnitude (∆F) (Lapole et al. 2023). The unifying view is that prolonged vibration decreases the activity of the MUs in the stimulated muscle. The primary spinal coordinator of agonist–antagonist muscle activity is disynaptic reciprocal inhibition through Ia inhibitory interneurons (Baldissera et al. 2011). The activation of Ia interneurons in parallel with motoneurons appears to be the mechanism by which antagonists may be inhibited while agonists are activated (Baldissera et al. 2011). These findings suggested that Ia firing from the biceps femoris muscle is reduced after vibration, resulting in reduced reciprocal inhibition and therefore reduced inhibition of VL muscle motoneurons. In this study, we found that vibration intervention for 30 s altered MU firing behavior (the discharge rate and recruitment threshold) and VL muscle activity (RMS) in the antagonist muscle of the muscle to which the vibration was applied, even though the required motor task was not changed. This finding provides new insight into how vibration stimulation can alter the neuromuscular regulatory mechanisms of antagonist muscles after intervention. However, a major limitation of this study was that it is unclear whether 30 s of vibration stimulation inhibited the activity of the vibrated muscle (the biceps femoris muscle) because this activity was not evaluated. This is a matter that can be investigated future research. A recent study provided new evidence that presynaptic mechanisms (Ia-α motoneuron synapses) are not involved in the depression of spinal excitability after local vibration (Souron et al. 2019). Souron et al. proposed that decreased motoneuron excitability could be related to reduced PIC strength in spinal motoneurons. In terms of motoneuron discharge characteristics, PICs play a critical role, as they amplify and prolong the effects of synaptic input (Heckmann et al. 2005). In recent years, several studies have been conducted using the ΔF method via HD-sEMG, and Lapole et al. reported that the PIC and discharge rate are altered during local vibration (Lapole et al. 2023). As this study did not include the derecruitment phase in the experimental motor task, the ΔF method cannot be used to estimate PIC. In the future, motor tasks can be modified to capture changes in the PIC in antagonist muscles after local vibration to investigate the effects on discharge rate in more detail. This issue can also be addressed in future research.

Although it was reported in a previous study that vibration of the antagonist muscle improved corticospinal excitability in the agonist muscle, as indicated by motor-evoked potential amplitudes elicited using transcranial magnetic stimulation (Forner-Cordero et al. 2008), we are the first to report a resultant increase in MU discharge rates, especially in the higher-threshold units. An increase in corticospinal excitability was shown to cause increased nervous system activity (Škarabot et al. 2018), which was shown to also affect the activity of MUs (Bawa and Lemon 1993). Corticospinal excitability has been reported to be affected by vibration frequency, with a frequency of approximately 75 Hz contributing the most to increased corticospinal excitability (Steyvers et al. 2003). Based on these findings, it is possible that the influence of corticospinal tracts may also play a role in the present results; however, the corticospinal response was not measured in this study, and thus, this cannot be confirmed at this time. In the future, the influence of the corticospinal tract should also be examined via the use of transcranial magnetic stimulation.

We found that the recruitment threshold is a factor that influences the discharge rate of MUs and that higher-threshold units are more strongly related to this parameter. In this study, it was confirmed that MUs that were recruited at higher thresholds had an increased discharge rate and a decreased recruitment threshold. Pollock et al. reported that whole-body vibration decreased the recruitment threshold of higher-threshold MUs and that higher- and lower-threshold MUs respond differently to vibration in healthy young adults (Pollock et al. 2012). These findings are in accordance with the results of the present study, which showed that the response was different for higher- and lower-threshold MUs. Romaiguère et al. reported that vibration increased Ia afferent discharges in young adults and concluded that the increase in the facilitatory action of Ia afferents on motoneurons might accompany voluntary supraspinal motoneuronal drive and thus lead to a postvibration reduction in the MU recruitment threshold (Romaiguère et al. 1993). These findings focus on the response of the vibrated muscle, and there are still few reports on antagonist muscles. The AVR could also be mediated by long loop reflexes via the cerebral cortex, which respond to somatosensory stimuli (Fetz et al. 1980; MacKinnon et al. 2000). These findings suggested that the antagonist muscle of the vibrated muscle is regulated at the cerebral level. The results of this study may be important for understanding the neural response in the antagonist muscle of the stimulated muscle.

It is widely known that local vibration contributes to muscle strengthening (Alghadir et al. 2018), and the factors that contribute to this phenomenon have been attributed to the excitability of the spinal cord (PICs) (Souron et al. 2019) and corticospinal pathways (Bawa and Lemon 1993; Škarabot et al. 2018). The reduced recruitment threshold and the increased discharge rate of MUs at the higher recruitment threshold could explain the greater effect on muscle strengthening. Although the discharge rate of MUs increased in this study, muscle strength did not change. Since we observed an increase in the discharge rate and RMS of the VL muscle, this finding suggested that it contributed to the activation of the MU firing behavior to a degree that did not affect muscle strength. One of the reasons for this difference in outcomes in this study was that muscle strength was assessed by maximal effort, whereas MU firing behavior was assessed during submaximal contractions (30% MVC) performed against a target force designed to maintain motor output. Even though it is desirable to assess MU firing behavior during maximal effort, the use of a surface electrode results in a reduction in the number of reliable MUs identified because the myoelectric signal becomes more complex and phase cancellation occurs more frequently (Keenan et al. 2005). We attempted to analyze MUs during MVC, but MUs could not be detected in most subjects. This was a limitation of the analysis method using HD-sEMG. The vibration conditions in this study were 30 s at an amplitude of 0.1 mm, and it is necessary to examine whether the effects of these conditions on muscle strength can be enhanced by changing parameters, such as by extending the vibration time or increasing the amplitude.

This study had several limitations. First, this study included only a 30% MVC task. It is not clear whether MUs recruited at nearly 50% or 60% MVC were affected more than MUs recruited at 10% to 30% MVC. To thoroughly examine the effects of vibration on MUs in more detail, it will be necessary to also include a high-intensity task (e.g., 60–80% MVC) in the study design. Second, this study only recruited males. In a previous study, sex differences were reported for the MU discharge rate and MU discharge rate variability due to tendon vibration (Harwood et al. 2014), and sex should be considered when examining the effect of vibration on MU firing behavior. Third, this study evaluated only the immediate effects of vibration intervention. It is necessary to conduct a long-term intervention study to determine whether vibration has a muscle strengthening effect on antagonist muscles. Fourth, this study targeted only one pair of agonist and antagonist muscles (the VL muscle and biceps femoris muscle). However, the effects of these interventions on other muscle pairs (e.g., the tibialis anterior muscle and triceps surae muscle) and on elderly people and females remain to be determined. Fifth, we acquired data only from the VL muscle. We considered the possibility that the small change in the discharge rate was responsible for the lack of increase in muscle strength in this study. However, it is also necessary to consider the possibility that the activation of the stimulated muscle (the biceps femoris muscle) or the inhibition of the activity of other quadriceps muscles (the rectus femoris and vastus medialis muscles) may have also been contributing factors to the above lack of increase in muscle strength. It will be necessary to include these muscles in future studies to determine the effects of vibration intervention on the stimulated and antagonist muscles in more detail. Finally, the motor task we used in this study did not include a derecruitment phase. The use of trapezoidal-type motor tasks, including recruitment and derecruitment phases, allows paired MU analysis (∆F method) (Stephenson and Maluf 2011; Nishikawa et al. 2022), which supports the estimated analysis of changes in PICs that are sensitive to excitatory synaptic input (Heckman et al. 2008). Future studies using additional and modified motor tasks, recruiting females and elderly people, including other muscle pairs, and considering long-term intervention are needed to elucidate the detailed influence of vibration on the firing behavior of MUs and the physical performance of the antagonist muscle. Furthermore, in this study, the intervention was carried out by applying the vibration transducer directly to the tendon, but to achieve commercialization, it is necessary to further examine the robustness of the intervention to determine whether a similar effect can be obtained even when the vibration transducer is applied over clothing.

## Conclusions

We demonstrated the effect of vibration on the firing behavior of MUs and the physical performance of the antagonist muscle in healthy young adults. Our results showed that vibration of the biceps femoris muscle increased the discharge rate and decreased the recruitment threshold of higher-threshold MUs in the VL muscle. These findings suggested that vibration has an immediate effect on the neural system controlling the antagonist muscle. These results support the future development of a seated vibration intervention to enhance strength training.

## Data Availability

Data are available from the corresponding author upon reasonable request.
